# New 5-adic Cantor sets and fractal string

**DOI:** 10.1186/2193-1801-2-654

**Published:** 2013-12-05

**Authors:** Ashish Kumar, Mamta Rani, Renu Chugh

**Affiliations:** Department of Mathematics, Maharshi Dayanand University, Rohtak, 124001 Haryana India; Department of Computer Science, Central University of Rajasthan, Rajasthan, 305801 Rajasthan India

**Keywords:** Cantor one-fifth set, p-adic integers, 5-adic Cantor one-fifth set, Iterated function system (IFS), Fractal string, 26A30, 28A80, 11E20, 26E30, 28E30, 26A80, 28A12

## Abstract

In the year (1879–1884), George Cantor coined few problems and consequences in the field of set theory. One of them was the Cantor ternary set as a classical example of fractals. In this paper, 5-adic Cantor one-fifth set as an example of fractal string have been introduced. Moreover, the applications of 5-adic Cantor one-fifth set in string theory have also been studied.

**Electronic supplementary material:**

The online version of this article (doi:10.1186/2193-1801-2-654) contains supplementary material, which is available to authorized users.

## Introduction

During the late eighteenth century, mathematicians delighted in producing sets with ever more weird properties, many of them now recognized to be fractal in nature (Crilly et al.). George Cantor (1879–1884) wrote a series of papers entitled “Uber unendliche lineare punktmannichfaltigkeiten” (Cantor 1879; 1880; 1882; 1883a; 1883b; 1884) that contained the first systematic treatment of the point set topology of real line, in which he triggered some problems and consequences in the field of set theory. One of these is the classical Cantor set problem devised by Cantor in the footnote to a statement saying that perfect sets do not need to be everywhere dense (Fleron 1994). In last two decades, Devil’s and other researchers established the graphical representation of Cantor sets in the form of staircases (Horiguchi and Morita 1984a; 1984b; Rani and Prasad 2010).

Middle one-third, a classical Cantor set found a celebrated place in the mathematical analysis and in its applications (Hutchinson 1981; Mendes 1999; Shaver 2010). For a fundamental work on Cantor set and its applications, one may refer to (Peitgen et al. 2004), (Devaney 1992), (Beardon 1965), (Falconer 1985), (Lapidus and van Frankenhuijsen 2006), (Gutfraind et al. 1990) and (Lee 1998). In recent years, p-adic analysis has been used in various areas of mathematics as well as in aspects of quantum physics and string theory (Lapidus and van Frankenhuijsen 2006). For a detailed analysis of fractal string and p-adic integers, one may refer to (Chistyakov 1996; Hung 2007; Koblitz 1984; Robert 2000; Schikhof 1984; Vladimirov et al. 1994).

Lapidus and van Frankenhuijsen (2000; 2006) introduced the concept of fractal string and established the geometric zeta function, zeros of zeta function, spectra of fractal string and the complex dimension of the fractal string. In 2008, (Lapidus 2008) suggested that fractal string and their quantization may be related to aspects of string theory. In last few decades, M. L. Lapidus, jointly with other researchers generalized and introduced the various properties of fractal string (see (Edgar 2008; Lapidus 1992; Lapidus and Maier 1995; Lapidus and Pearse 2006; 2008; Lapidus and Pomerance 1993)).

In 2008, (Lapidus and Hung 2008; 2009) provided a framework for unifying the archimedean and p-adic (nonarchimedean) fractal string with their geometric zeta functions and complex dimensions for 3-adic Cantor sets and also the general case for p-adic Cantor sets respectively. Recently, (Ashish, Mamta Rani and Renu Chugh, Variants of Cantor Sets Using IFS, submitted and Ashish, Mamta Rani and Renu Chugh, Study of Variants of Cantor sets., submitted) studied the variants of Cantor sets and established their mathematical analysis using mathematical feedback system and iterated function system respectively.

Our goal in this paper is to study the Cantor one-fifth set as a new classical example of fractal string. Moreover, the non-archimedean (5-adic) Cantor one-fifth set with their applications in string theory has also been established. In the third section, the main results of our study have been presented, followed by the “Concluding remarks” section.

## Preliminaries

In this section, we recall some basic definitions pertaining to the notion of (ordinary) fractal string and introduce several new ones such as the most important of which are quinary expansion and Cantor one-fifth set:

### Definition 2.1. Cantor one-fifth set

The Cantor one-fifth set for unequal intervals is defined as the *F* = ∩ *F*_*n*+1_, where *F*_*n*+1_ is constructed by dividing *F*_*n*_ in five unequal line segments and removing second and fourth one-fifth line segment, *F*_0_ being the closed interval 0 ≤ *x* ≤ 1 (Ashish, Mamta Rani and Renu Chugh, Variants of Cantor Sets Using IFS, submitted).

### Definition 2.2. Quinary expansion

The sequence 0.*x*_1_*x*_2_*x*_3_*x*_4_*x*_5_…, where each *x*_*i*_ is either 0, 1, 2, 3, or 4 is called quinary expansion of *x* if *x* = *x*_1_/5 + *x*_2_/5^2^ + *x*_3_/5^3^ + ....

For example, the sequence 0.04444… is the quinary expansion of 1/5 since we have

$$\frac{0}{5}+\frac{4}{5^{2}}+\frac{4}{5^{3}}+\frac{4}{5^{4}}+\frac{4}{5^{5}}+\frac{4}{5^{6}}+......=\frac{4}{5^{2}}\sum_{i=0}^{\infty }\frac{1}{5^{i}}=\frac{1}{5}$$

Lapidus and van Frankenhuijsen (2000) and (2006), introduced the concept of fractal strings as follows:

### Definition 2.3. Fractal string

A fractal string Ω is a bounded open subset of the real line *R*. The collection of lengths ℓ_*j*_ of the disjoint intervals is denoted by *L*.

For example, the complement of the Cantor set in the closed unit interval [0, 1] is a Cantor string. Moreover, the topological boundary of Cantor string is the Cantor set *C* itself.

### Definition 2.4. Geometric zeta function

The geometric zeta function of a fractal string Ω with lengths *L* is

$$\varsigma_{L}\left(s\right)=\sum_{k=1}^{\infty }m_{k}\ell_{k}^{s}$$

where ℓ_1_, ℓ_2_, …, ℓ_*k*_ are the lengths of open intervals and *m*_*k*_ be the corresponding multiplicity of open intervals (Lapidus and van Frankenhuijsen 2000).

For example, Cantor string consists of intervals of lengths ℓ_1_ = (*l*_1_ = 1/3), ℓ_2_ = (*l*_2_ = *l*_3_ = 1/9), ℓ_3_ = (*l*_4_ = *l*_5_ = *l*_6_ = *l*_7_ = 1/27), and so on, that is, the lengths are the numbers 3^-*k*-1^ with multiplicity $m_{3^{-k-1}}=2^{k}$ for *k* = 0, 1, 2, 3, …. . So, the geometric zeta function is:

ςLs=∑k=0∞mkℓks=∑k=0∞2k.3-k-1s=3-s1-2.3-sforRes>D

where D = log2/log3 is the dimension of usual Cantor set.

Recently, (Ashish, Mamta Rani and Renu Chugh, Variants of Cantor Sets Using IFS, submitted), established the self-similarity of the Cantor one-fifth set using the iteration function system as follows:

### Theorem 2.1

Let *f*_1_, *f*_2_ and *f*_3_ be the similarity contraction mappings on *ℝ* defined by

$$\begin{array}{ccc} f_{1}\left(x\right)=x/5, & f_{2}\left(x\right)=(x+2)/5, & f_{3}\left(x\right)=(x+4)/5, \end{array}$$

where all the mappings have the ratio 1/5. Then, the Cantor one-fifth set *F* satisfies the self-referential equation

$$F=f_{1}\left[F\right]\cup f_{2}\left[F\right]\cup f_{3}\left[F\right]$$

for the iterated function system (*f*_1_, *f*_2_, *f*_3_).

## Main results

### 5-adic (nonarchimedean) Cantor one-fifth set

A sequence (*s*_*i*_)_*i* ∈ *ℕ*_ of natural numbers between 0 and *p*-1 (inclusive) is a *p*-adic integer. We write this conventionally as .....*s*_*i*_.....*s*_2_ *s*_1_ *s*_0_. If ‘*n*’ is any natural number, and

$$n=\frac{}{s_{k-1}\;s_{k-2}....\;s_{1}\;s_{0}\;}$$

is its *p*-adic representation (in other words, $n=\sum_{i=0}^{k-1}s_{i}p^{i}$ with each *s*_*i*_ is a *p*-adic digit), then we identify ‘*n*’ with the *p*-adic integer (*s*_*i*_) with *s*_*i*_ = 0 if *i* ≥ *k* (Madore 2000). Further, the set of *p*-adic integers, which we call *ℤ*_*p*_ with two binary operations on it (addition and multiplication) is a ring. The relation between the set (ring) *ℤ*_*p*_ of *p*-adic integers and the set (field) *ℚ*_*p*_ of *p*-adic numbers is the same as between the set (ring) *ℤ* of integers and the set (field) *ℚ* of rationals (Madore 2000). Since, *ℤ*_*p*_ is an important subspace of *ℚ*_*p*_, it can be represented as follows:

$$\;\begin{array}{cc} {\mathbb{Z}}_{p}=\{s_{0}+s_{1}p^{1}+s_{2}p^{2}+\ldots ;s_{i}\in \left(0,1,2,\ldots ,p-1\right), & \mathrm{for}\;\mathrm{all}\;i\geq 0\} \end{array}$$

For this *p*-adic expansion, we can also write

$${\mathbb{Z}}_{p}=\overset{p-1}{\underset{c=0}{\cup }}\left(c+p{\mathbb{Z}}_{p}\right),$$

where *c* + *pℤ*_*p*_ = {*y* ∈ *ℚ*_*p*_ : |*y* - *c*|_*p*_ ≤ 1/*p*} (Lapidus and van Frankenhuijsen 2006) It is also known that there are topological models of *ℤ*_*p*_ in the Euclidean space *ℝ*^*d*^ as fractal spaces such as the Cantor set and the Sierpinsky gasket (Robert 2000), where *ℤ*_*p*_ is homeomorphic to the ternary Cantor set. Now, we consider the ring of 5-adic integers *ℤ*_5_, that is, homeomorphic to Cantor one-fifth set.

**Figure 1:**
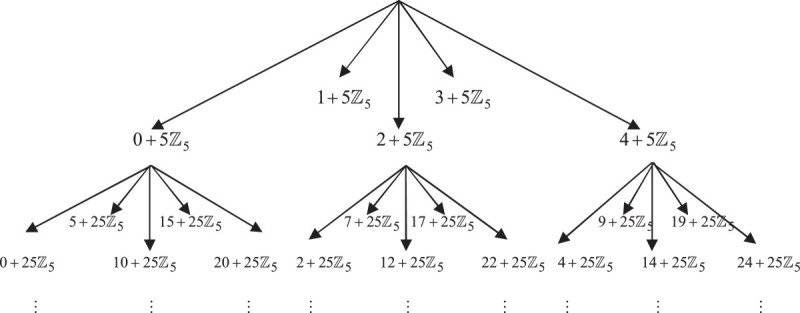
**5-adic (nonarchimedean) Cantor one-fifth set.**

Figure 1 below shows the representation of 5-adic Cantor one-fifth set ‘*N*’. To start the construction, initiator *N*_0_ = *ℤ*_5_ is subdivided into five equal subintervals 0 + 5*ℤ*_5_, 1 + 5*ℤ*_5_, 2 + 5*ℤ*_5_, 3 + 5*ℤ*_5_ and 4 + 5*ℤ*_5_. Drop the subintervals 1 + 5*ℤ*_5_ and 3 + 5*ℤ*_5_ and repeat the same process for the remaining subintervals. Further, repeating the same process over and over again, by removing the open subintervals of second and fourth position at each step from each closed interval, we obtain a sequence *N*_*k*_ for *k* = 1, 2, . . . The 5-adic Cantor one-fifth set (see Figure 1) *N*_*k*_ consists of 3^*k*^ disjoint closed intervals. Thus, the 5-adic Cantor one-fifth set would be the limit ‘*N*’ of the sequence *N*_*k*_ of sets. So, we define limit ‘*N*’ as the intersection of the sets *N*_*k*_ i.e.

$$N=\underset{k\in \mathbb{N}}{\cap }N_{k}.$$

#### Theorem 3.1

Let *f*_1_, *f*_2_ and *f*_3_ be the similarity contraction mappings on 5-adic integer *ℤ*_5_ defined by

1 $$\begin{array}{ccc} f_{1}\left(x\right)=5x, & f_{2}\left(x\right)=5x+2, & f_{3}\left(x\right)=5x+4, \end{array}$$

with scaling ratio 1/5. Then, the 5-adic Cantor one-fifth set *N* satisfies the self-referential equation

2 $$N=f_{1}\left[N\right]\cup f_{2}\left[N\right]\cup f_{3}\left[N\right].$$

Proof: Using above construction of 5-adic Cantor one-fifth set, we can say that

$$N_{k+1}=f_{1}\left[N_{k}\right]\cup f_{2}\left[N_{k}\right]\cup f_{3}\left[N_{k}\right]$$

for all *k* ≥ 1. Since, the mapping *f*_*j*_ for *j* = 1, 2, 3 is one-to-one and *N* = ∩ *N*_*k*_, then it implies that

*f*_*j*_[*N*] = *f*_*j*_[ ∩ *N*_*k*_] = ∩ *f*_*j*_[*N*_*k*_], for *k* = 1, 2, ….

so that, we can write *f*_1_[*N*] = ∩ *f*_1_[*N*_*k*_], *f*_2_[*N*] = ∩ *f*_2_[*N*_*k*_]and *f*_3_[*N*] = ∩ *f*_3_[*N*_*k*_],

therefore, *f*_1_[*N*] ∪ *f*_2_[*N*] ∪ *f*_3_[*N*] = ( ∩ *f*_1_[*N*_*k*_]) ∪ ( ∩ *f*_2_[*N*_*k*_]) ∪ ( ∩ *f*_3_[*N*_*k*_])

$$f_{1}\left[N\right]\cup f_{2}\left[N\right]\cup f_{3}\left[N\right]=\cap \left(f_{1}\left[N_{k}\right]\cup f_{2}\left[N_{k}\right]\cup f_{3}\left[N_{k}\right]\right)$$

$$f_{1}\left[N\right]\cup f_{2}\left[N\right]\cup f_{3}\left[N\right]=\cap N_{k+1}=N$$

$$f_{1}\left[N\right]\cup f_{2}\left[N\right]\cup f_{3}\left[N\right]=N$$

which gives the proof of the theorem.

**Figure 2:**
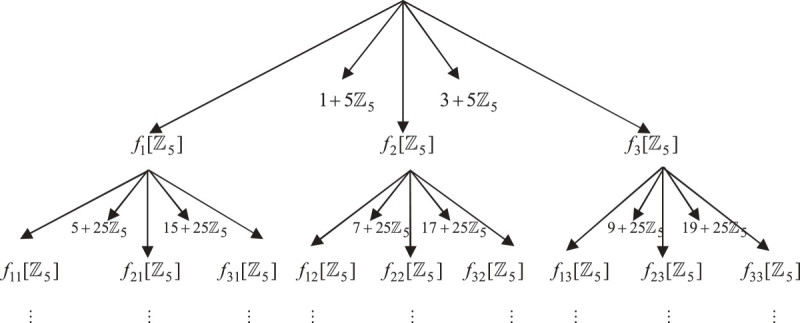
**5-adic Cantor one-fifth set using IFS.**

Figure 2 shows the graphical representation of 5-adic Cantor one-fifth set using iterated function system (*f*_1_, *f*_2_, *f*_3_).

### Quinary expansion of 5-adic Cantor one-fifth set

#### Theorem 3.2

The 5-adic Cantor one-fifth set is represented by the quinary expansion of its elements in the form

3 $$N=\left\{x\in {\mathbb{Z}}_{5}:x=x_{0}+5^{1}x_{1}+5^{2}x_{2}+\ldots ,x_{j}\in \left\{0,2,4\right\}\;\right\}$$

for all *j* = 0, 1, 2, .....

Proof: Let us define the inverse of similarity contraction mappings *f*_1_, *f*_2_ and *f*_3_, on *ℤ*_5_ as follows:

4 $$\begin{array}{ccc} f_{1}^{-1}\left(x\right)=x/5, & f_{2}^{-1}\left(x\right)=(x-2)/5, & f_{3}^{-1}\left(x\right)=(x-4)/5, \end{array}$$

Now, for *x*_*j*_ ∊ {0, 1, 2, 3, 4}, for all *j* ≥ 0, either

5 $$\;\begin{array}{ccc} x=x_{0}+5^{1}x_{1}+5^{2}x_{2}+\ldots ,\in 1+5{\mathbb{Z}}_{5} & \mathrm{or} & 3+5{\mathbb{Z}}_{5}, \end{array}$$

if and only if either *x*_0_ = 1 or *x*_0_ = 3, respectively. Let *η*, *μ* ∈ *ℕ* be the fixed subscript numbers such that *x*_*η*_ = 1and *x*_*μ*_ = 3. Thus, *x*_*j*_ = 0, 2 or 4, for all *j* > *η* and all *j* > *μ*. Since, we have divided the real line into five equal line segments denoted by 0, 1, 2, 3, and 4 respectively. Thus, if *x*_0_ = 0, then we use the function *f*_1_^-1^ for all *x* ∊ *N*, if *x*_0_ = 2, then use the function *f*_2_^-1^ for all *x* ∊ *N* and if *x*_0_ = 4, then use the function *f*_3_^-1^ for all *x* ∊ *N*. Thus, from these three cases, we obtain

$$\begin{array}{l} f_{1}^{-1}\left(x\right)=f_{2}^{-1}(x)=f_{3}^{-1}(x)=x_{1}+5^{1}x_{2}+\ldots ,+5^{\eta -1}x_{\eta }+5^{\eta }x_{\eta +1}+\ldots ,\\ f_{1}^{-1}\left(x\right)=f_{2}^{-1}(x)=f_{3}^{-1}(x)=x_{1}+5^{1}x_{2}+\ldots ,+5^{\mu -1}x_{\mu }+5^{\mu }x_{\mu +1}+\ldots \end{array}$$

again repeating the process in this manner, we obtain the general case

$$\begin{array}{l} f_{1}^{-1}\left(x\right)=f_{2}^{-1}(x)=f_{3}^{-1}(x)=x_{\eta }+5x_{\eta +1}+\ldots ,\\ f_{1}^{-1}\left(x\right)=f_{2}^{-1}(x)=f_{3}^{-1}(x)=x_{\mu }+5x_{\mu +1}+\ldots \end{array}$$

which lie in the intervals 1 + 5*ℤ*_5_  and 3 + 5*ℤ*_5_  respectively. Thus, we found that

$$\begin{array}{ccc} N\cap \left(1+5{\mathbb{Z}}_{5}\right)=\emptyset & \mathrm{and} & N\cap \left(3+5{\mathbb{Z}}_{5}\right)=\emptyset \end{array}$$

Hence either *x* ∈ 1 + 5*ℤ*_5_  or *x* ∈ 3 + 5*ℤ*_5_  which deduce that *x* ∉ *N*. Hence we proved that for *x*_*j*_ ∊ {0, 2, 4}, *x* ∊ *N*.

Conversely, let all the variables *x* = *x*_0_ + 5^1^*x*_1_ + 5^2^*x*_2_ + …, belong to *ℤ*_5_ for all *x*_*j*_ ∊ {0, 2, 4}, and *j* = 0, 1, 2, …. Then, from Eq. (3) and (5), we can say that neither *x* ∈ 1 + 5*ℤ*_5_  nor *x* ∈ 3 + 5*ℤ*_5_  which implies that *x* ∉ *f*_*j*_(1 + 5*ℤ*_5_) and also *x* ∉ *f*_*j*_(3 + 5*ℤ*_5_), for *j* ∊ *W*_*l*_ = {1, 2, 3}^*l*^, *l* = 0, 1, 2, ..... Thus,

$$x\notin \left\{\left(\overset{\infty }{\underset{l=0}{\cup }}\underset{j\in W_{l}}{\cup }f_{j}\left(1+5{\mathbb{Z}}_{5}\right)\right)\cup \left(\overset{\infty }{\underset{l=0}{\cup }}\underset{j\in W_{l}}{\cup }f_{j}\left(3+5{\mathbb{Z}}_{5}\right)\right)\right\}=Y$$

Thus, *N* ∪ *Y* = *ℤ*_5_ and hence *x* ∊ *N*, which completes the proof of the theorem.

### Cantor one-fifth set as fractal string

It is well known from the definition of fractal string that such a set consists of countably many disjoint open intervals. The lengths of which form a sequence *L* = ℓ_1_, ℓ_2_, ℓ_3_, …, called the lengths of the string. We can assume without loss of generality that

$$\ell_{1}\geq \ell_{2}\geq \ell_{3},\ldots ,>0$$

where each length is counted according to its multiplicity. An ordinary fractal string can be thought of as a one-dimensional drum with fractal boundary. In the literature of fractal geometry, we found a classical example of the fractal string as Cantor string. It is the set, complement of the interval [0, 1] of the usual ternary Cantor set. It is one of the simplest and most important example in the research of fractal string by (Lapidus and van Frankenhuijsen 2006). Information about the geometry of Cantor string like Minkowski dimension and the Minkowski measurability is obtained from its geometric zeta function. Motivated by the research of Lapidus with other researcher’s (Lapidus and Hung 2008) on the Cantor string, we introduce a new Cantor one-fifth set as an example of fractal string.

**Figure 3:**
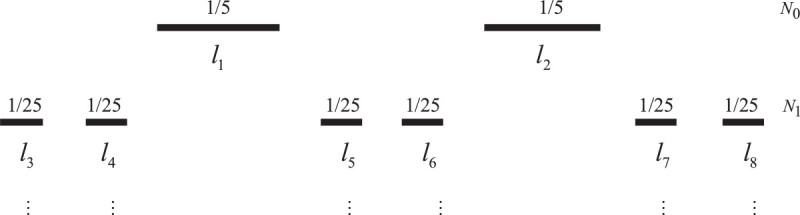
**Cantor one-fifth set as fractal string.**

The Cantor one-fifth string ℵ, is the complement of [0, 1] of the usual Cantor one-fifth set *F*. The Figure 3 shows the geometrical representation of Cantor one-fifth string.

Thus, we obtain

$$\begin{array}{l} ℵ=\left(1/5,2/5\right)\cup (3/5,4/5)\cup (1/25,2/25)\cup (3/25,4/25)\cup \\ (11/25,12/25)\cup \left(1/25,2/25\right)\cup (13/25,14/25)\cup \\ (21/25,22/25)\cup (23/25,24/25)\cup p \end{array}$$

where, ℓ_1_ = (*l*_1_ = *l*_2_ = 1/5), ℓ_2_ = (*l*_3_ = *l*_4_ = *l*_5_ = *l*_6_ = *l*_7_ = *l*_8_ = 1/25) and so on. Continuing in this way, we find that the lengths of open intervals is consist of ℓ_*k*_ = 5^-*k*-1^ with multiplicity $m_{5^{-k-1}}=2.3^{k}$ for *k* = 0, 1, 2, ....

Thus, the geometric zeta function of the Cantor one-fifth string is determined by the sequence ℵ:

6 ςℵs=∑k=0∞mkℓks=∑k=0∞2.3k.5-k-1s=2.5s-15s-3forRes>log3/log5

The poles of the such function are the set of complex numbers (see (Lapidus and Hung 2008), pp. 7) and given by

7 $$D_{L}=\left\{D+\mathrm{inp}:n\in \mathbb{Z}\right\},=\left\{0.6826+\mathrm{in}2\pi /\log5:n\in \mathbb{Z}\right\},$$

where *D* = log 3/log 5 = 0.6826 is the dimension of Cantor one-fifth set and *p* = 2*π*/log 5 oscillatory period of Cantor one-fifth string ℵ, is called *complex dimension* of Cantor one-fifth string.

**Figure 4:**
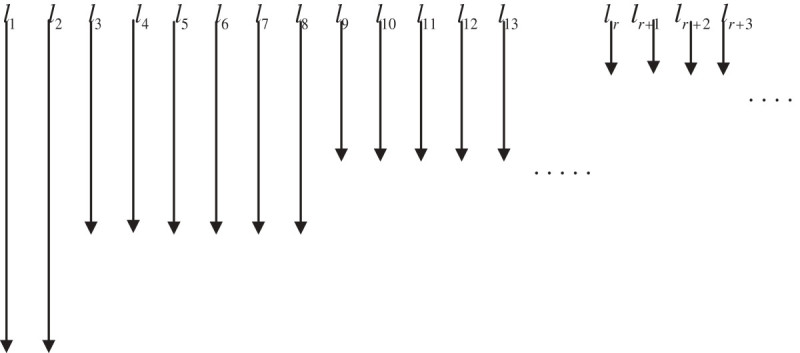
**Fractal harp of Cantor one-fifth string.**

Further, representation of Cantor one-fifth string may be seen in Figure 4 using fractal harp.

### 5-adic Cantor one-fifth set as fractal string

Since, the construction of 5-adic Cantor one-fifth string (*ξ*) is analogue to the usual Cantor one-fifth set. We start, by subdividing the interval *ℤ*_5_ into closed subintervals

$$\begin{array}{c} f_{1}\left({\mathbb{Z}}_{5}\right)=0+5{\mathbb{Z}}_{5}\\ f_{2}\left({\mathbb{Z}}_{5}\right)=2+5{\mathbb{Z}}_{5}\\ f_{3}\left({\mathbb{Z}}_{5}\right)=4+5{\mathbb{Z}}_{5} \end{array}$$

since, fractal string is complement of the usual Cantor one-fifth set in the closed interval [0, 1], the remaining open subintervals after this step are given by

$$\begin{array}{l} {\mathbb{Z}}_{5}-\overset{2}{\underset{j=1}{\cup }}f_{j}\left({\mathbb{Z}}_{5}\right)=1+5{\mathbb{Z}}_{5}=G_{1},\\ {\mathbb{Z}}_{5}-\overset{3}{\underset{j=2}{\cup }}f_{j}\left({\mathbb{Z}}_{5}\right)=3+5{\mathbb{Z}}_{5}=G_{2} \end{array}$$

then, the *G*_1_ ∪ *G*_2_ is the first sub-ring of self similar 5-adic Cantor one-fifth string. The lengths of *G*_1_ and *G*_2_ are given by using the Haar measure (Gupta and Jain 1986) as follows:

$$l_{1}=l_{2}=\mu_{H}\left(G_{1}\right)=\mu_{H}\left(G_{2}\right)=1/5$$

Again repeating the same process, by subdividing the closed intervals of first step (see Figure 1), we get

$$\begin{array}{l} f_{11}\left[{\mathbb{Z}}_{5}\right]=0+25{\mathbb{Z}}_{5},\;f_{21}\left[{\mathbb{Z}}_{5}\right]=10+25{\mathbb{Z}}_{5},\\ f_{31}\left[{\mathbb{Z}}_{5}\right]=20+25{\mathbb{Z}}_{5},\;f_{12}\left[{\mathbb{Z}}_{5}\right]=2+25{\mathbb{Z}}_{5},\\ f_{22}\left[{\mathbb{Z}}_{5}\right]=12+25{\mathbb{Z}}_{5},\;f_{32}\left[{\mathbb{Z}}_{5}\right]=22+25{\mathbb{Z}}_{5},\\ f_{13}\left[{\mathbb{Z}}_{5}\right]=4+25{\mathbb{Z}}_{5},\;f_{23}\left[{\mathbb{Z}}_{5}\right]=14+25{\mathbb{Z}}_{5},\\ f_{33}\left[{\mathbb{Z}}_{5}\right]=24+25{\mathbb{Z}}_{5}. \end{array}$$

Thus, the remaining open subintervals are given by

$$\begin{array}{ll} {\mathbb{Z}}_{5}-\overset{2}{\underset{j=1}{\cup }}f_{j1}\left({\mathbb{Z}}_{5}\right)=5+25{\mathbb{Z}}_{5}=G_{3}, & {\mathbb{Z}}_{5}-\overset{3}{\underset{j=2}{\cup }}f_{j1}\left({\mathbb{Z}}_{5}\right)=15+25{\mathbb{Z}}_{5}=G_{4},\\ {\mathbb{Z}}_{5}-\overset{2}{\underset{j=1}{\cup }}f_{j2}\left({\mathbb{Z}}_{5}\right)=7+25{\mathbb{Z}}_{5}=G_{5}, & {\mathbb{Z}}_{5}-\overset{3}{\underset{j=2}{\cup }}f_{j2}\left({\mathbb{Z}}_{5}\right)=17+25{\mathbb{Z}}_{5}=G_{6},\\ {\mathbb{Z}}_{5}-\overset{2}{\underset{j=1}{\cup }}f_{j3}\left({\mathbb{Z}}_{5}\right)=9+25{\mathbb{Z}}_{5}=G_{7}, & {\mathbb{Z}}_{5}-\overset{3}{\underset{j=2}{\cup }}f_{j3}\left({\mathbb{Z}}_{5}\right)=19+25{\mathbb{Z}}_{5}=G_{8}. \end{array}$$

The subring *G*_3_ ∪ *G*_4_ ∪ *G*_5_ ∪ *G*_6_ ∪ *G*_7_ ∪ *G*_8_is the second set of self-similar 5-adic Cantor one-fifth string. Thus, the length is given by

$$\begin{array}{l} l_{3}=l_{4}=l_{5}=l_{6}=l_{7}=l_{8}=\mu_{H}\left(G_{3}\right)=\mu_{H}\left(G_{4}\right)\\ \;=\mu_{H}\left(G_{5}\right)=\mu_{H}\left(G_{6}\right)=\mu_{H}\left(G_{7}\right)=\mu_{H}\left(G_{8}\right)=1/25. \end{array}$$

**Figure 5:**
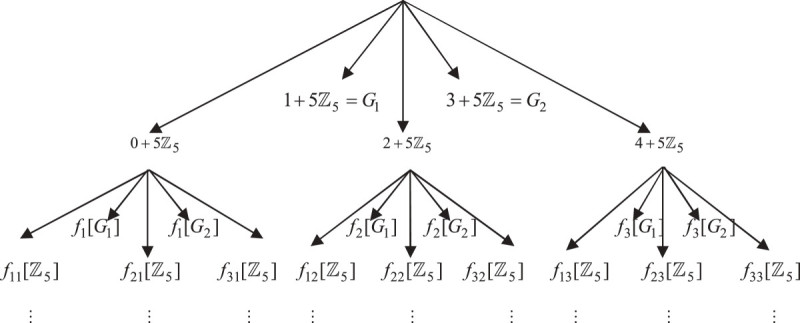
**5-adic Cantor one fifth string via IFS.**

Repeating the same process over and over again, we obtain a sequence ℓ_1_ = ℓ_2_ = ℓ_3_ = ℓ_4_ = ℓ_5_ = ..... which consists of lengths 5^-*k*-1^ with multiplicity 2.3^*k*^. Using Figure 5 the 5-adic Cantor one-fifth string can also be written as follows:

$$\begin{array}{l} \xi =\left(1+5{\mathbb{Z}}_{5}\right)\cup (3+5{\mathbb{Z}}_{5})\cup (5+25{\mathbb{Z}}_{5})\cup (15+25{\mathbb{Z}}_{5})\cup \\ \left(7+25{\mathbb{Z}}_{5}\right)\cup (17+25{\mathbb{Z}}_{5})\cup (9+25{\mathbb{Z}}_{5})\cup (19+25{\mathbb{Z}}_{5})\cup .... \end{array}$$

From Definition 2.3 (Lapidus and Hung 2009), the geometric zeta function of *ξ* is given by

8 ςξ=μH1+5ℤ5s+μH3+5ℤ5s+μH5+25ℤ5s+…=∑k=1∞mkℓks=∑k=1∞2.3k.5-k-1s=2.5s-15s-3forRes>log3/log5

the poles of the such function are the set of complex numbers

9 $$D_{L}=\left\{D+\mathrm{inp}:n\in \mathbb{Z}\right\}=\frac{\log3}{\log5}+\mathrm{in}\frac{2\pi }{\log5},$$

where *D* = log 3/log 5 = 0.6826 is the dimension of 5-adic Cantor one-fifth string and *p* = 2*π*/log 5 oscillatory period is the volume of the inner tubular neighborhood of *ξ*.

## Concluding remarks

Based on the results, our conclusions are following:

1. In Subsection “5-adic (nonarchimedean) Cantor one-fifth set”, using 5-adic integer it has been concluded that Cantor one-fifth set satisfies the nonarchimedean properties of a set and also studied that nonarchimedean Cantor one-fifth set satisfies self-similarity property using self-referential equation.
2. Further, it has been concluded that quinary Cantor one-fifth set is homeomorphic to 5-adic Cantor one-fifth set *N* in subsection “Quinary expansion of 5-adic Cantor one-fifth set”.
3. In Subsection “Cantor one-fifth set as fractal string” and “5-adic Cantor one-fifth set as fractal string”, it has been analyzed that Cantor one-fifth set and 5-adic Cantor one-fifth set both satisfy the properties of fractal string. Moreover, we found that the geometric zeta function and the complex dimension of both the sets are perfectly same.

## Authors’ original submitted files for images

Below are the links to the authors’ original submitted files for images. Authors’ original file for figure 1 Authors’ original file for figure 2 Authors’ original file for figure 3 Authors’ original file for figure 4 Authors’ original file for figure 5
