# Supplementary figures and images for: New 5-adic Cantor sets and fractal string
Source: Springerplus. 2013 Dec 5;2(1):654. doi: 10.1186/2193-1801-2-654 (PMC3872287; doi:10.1186/2193-1801-2-654)

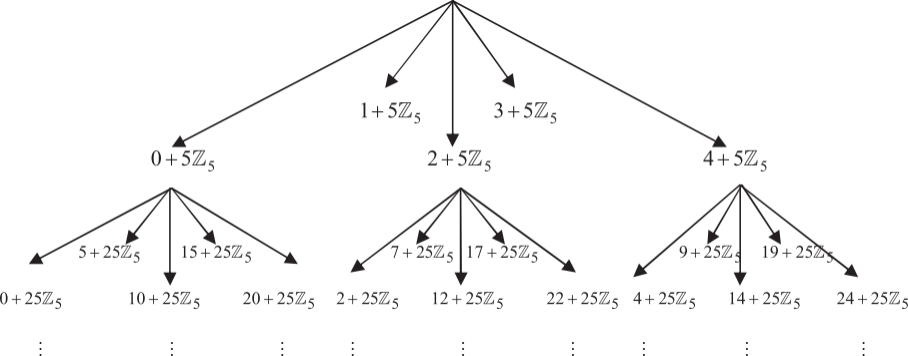

Supplement: Supplementary file 1 — Authors’ original file for figure 1 [file 40064_2013_741_MOESM1_ESM.pdf]

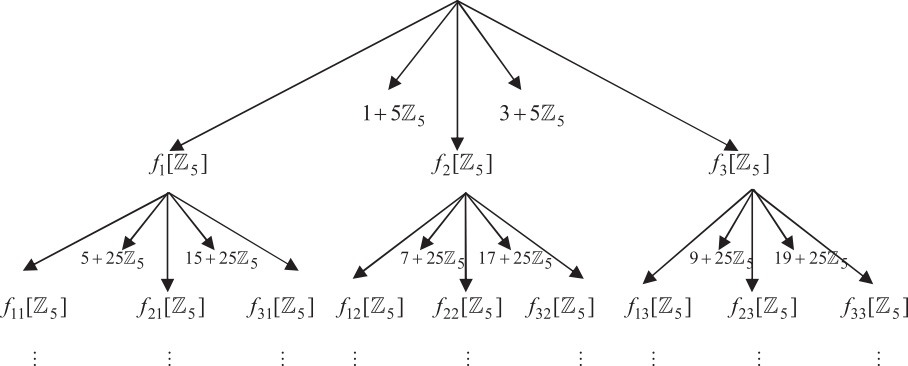

Supplement: Supplementary file 2 — Authors’ original file for figure 2 [file 40064_2013_741_MOESM2_ESM.pdf]

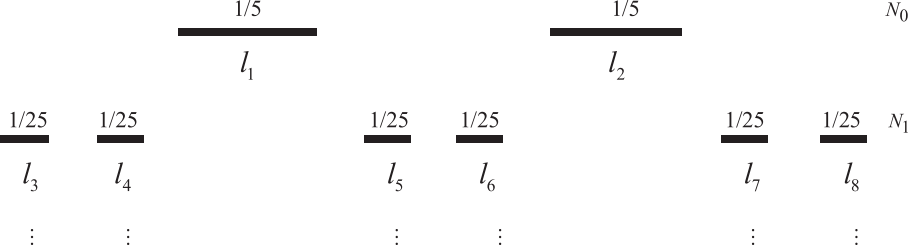

Supplement: Supplementary file 3 — Authors’ original file for figure 3 [file 40064_2013_741_MOESM3_ESM.pdf]

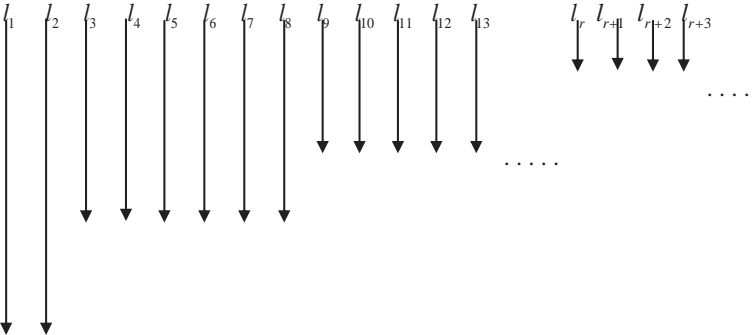

Supplement: Supplementary file 4 — Authors’ original file for figure 4 [file 40064_2013_741_MOESM4_ESM.pdf]

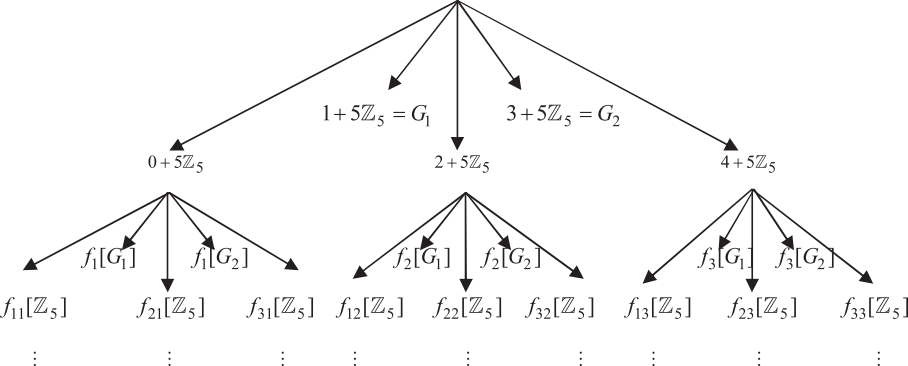

Supplement: Supplementary file 5 — Authors’ original file for figure 5 [file 40064_2013_741_MOESM5_ESM.pdf]
